# Competition drives the dispersal dynamics of two cup coral morphs in populations on the Powell Basin slopes, Weddell Sea, Antarctica

**DOI:** 10.1038/s41598-025-02282-7

**Published:** 2025-05-24

**Authors:** Tasnuva Ming Khan, Huw J. Griffiths, Nile P. Stephenson, Rowan J. Whittle, Autun Purser, Andrea Manica, Emily G. Mitchell

**Affiliations:** 1 Department of Zoology, University of Cambridge, Downing St, Cambridge, CB2 3EJ UK; 2 British Antarctic Survey, High Cross, Madingley Rd, Cambridge, CB3 0ET UK; 3 University Museum of Zoology Cambridge, New Museums Site, Downing Street, Cambridge, CB2 3EJ UK; 4 Alfred Wegener Institute, Helmholtz Centre for Marine and Polar Research, Am Handelshafen 12, 23570 Bremerhaven, Germany

**Keywords:** Competitive coexistence, Dispersal dynamics, Spatial analyses, Antarctica, Cup corals, Alpha diversity, Community ecology, Population dynamics

## Abstract

Coexistence of ecologically similar taxa can contribute considerably to local biodiversity patterns. Deep water Southern Ocean benthic communities provide a unique setting to investigate coexistence mechanisms due to the relatively pristine nature of Antarctic ecosystems and a lack of disturbances like ice scour or top-down predator control. Here, we examine cup coral populations on the deep (~ 2000 m) rocky slopes of Powell Basin, Weddell Sea—an ecosystem with dense and speciose epibenthic communities. We investigate the spatial ecology of two coral morphotypes—“orange” and “pink” cup corals (likely *Caryophyllia* or *Flabellum*) using high-resolution seabed images from the RV *Polarstern* cruise PS118. Across 36 sites, we recorded 3431 pink and 1545 orange corals, which formed both mixed and single-population dominant (where either morph was near absent) communities. Spatial point process analysis revealed that reproductive processes drive their spatial patterns, with orange corals showing consistent dispersal behaviour regardless of community type. In contrast, pink corals exhibited greater dispersal plasticity in mixed populations, significantly increasing dispersal distances, suggesting that they are the weaker competitors. Our results suggest that in these deep water hard substrate Antarctic communities, dispersal plasticity has the ability to enable coexistence of ecologically similar morphs, thereby increasing alpha diversity.

## Introduction

Competition among biota is a fundamental structuring force of communities, where one possible outcome of interspecific competition is that individuals of one species suffer a reduction in growth, fecundity or survivorship due to exploitation of resources by individuals of another species^1^. The classic Lotka–Volterra model of interspecific competition^2,3^ suggests that two species with similar ecological niches cannot coexist indefinitely. A common outcome of this limitation is that one species may outcompete and exclude another from a habitat entirely. Such instances of competitive exclusion by similar species has been observed in a suite of terrestrial and marine environments, such as in the altitudinal separation of salmonid fishes in freshwater streams^4^, the total exclusion of one bedstraw plant species depending on soil type^5^, and the spatial segregation of barnacles in intertidal zones^6^. Competition for space, leading to (near) exclusion, has also been documented in extreme conditions, such as under the ice shelves of Antarctica, where cheilostome bryozoans outcompete cyclostome bryozoans for hard substrates^7^. However, coexistence of species with similar ecological function is also common, as coexistence via niche differentiation or stabilizing forces allows for high species richness^8^.

Several processes allow for the coexistence of apparently very similar organisms. For example, in coral reefs, niche differentiation allows for high cryptic diversity as sibling scleractinian species occupy distinct ecological niches^9,10^. This niche partitioning is observed within corals as well as in their algal symbionts^11^. External forces also allow for coexistence of ecologically similar taxa by exerting selection pressures, which reduces population sizes of the dominant taxa, enabling less competitive species to also thrive. In reefs, forces such as grazing and predation^12–14^ cause phase shifts between hard coral and macroalgal dominance^13,14^. These instances of intermediate disturbance^15–17^, whether by predator–prey interactions or events like storms^16,18,19^, maintain high alpha diversity. However, coexistence of species with similar function and morphology may also entirely be due to neutral effects^20^.

The effects of reproductive dispersal processes are scale-dependent^21^, with processes at the local and short term level mediating colonization of habitats, community assembly, and preventing local extinctions, thereby maintaining local alpha diversity^22,23^. At the macroecological scale, the mode of dispersal (such as brooding versus broadcast spawning) is a key factor that can shape patterns of biogeography for sessile taxa, mediating their biogeographic ranges^21^. On longer temporal and larger spatial scales, higher levels of within or between species dispersal can lead to declines in between-community (beta) and regional (gamma) diversity, as the metacommunities increasingly homogenize due to the strongest competitors excluding other species and impeding speciation^24^.

**Fig. 1:**
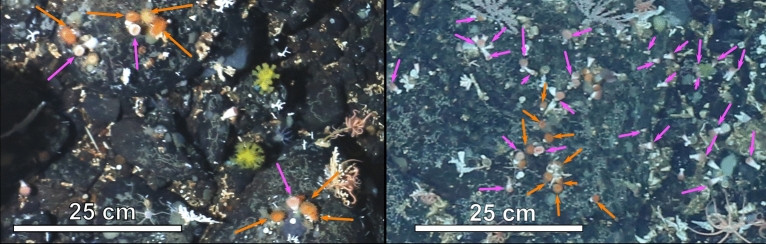
Cup corals in this study. Two cup coral morphotypes are present in the Powell Basin: the orange morph and the pink morph. Orange arrows point to orange morphs, pink arrows point to the pink morph.

Southern Ocean benthic marine communities provide a unique setting within which to investigate ecosystem structure and processes, such as coexistence mechanisms, as they are ecosystems with relatively little anthropogenic activity and low durophagous (i.e. consumption of prey with exoskeletons) predation rates^25,26^. Hard substrate slope communities on the Powell Basin in the Weddell Sea, Antarctica, support highly abundant epibenthic communities, with photographic surveys identifying over 50 different morphotaxa^27^, including cup corals, which are present as two different morphotypes^27^ (Fig. 1). These two morphs provide an opportunity to investigate how apparently morphologically very similar taxa adapt to competition since they exist both in isolation and together^27^, and thus have the potential to shed light on coexistence mechanisms. In an ecosystem where there is lack of intermediate disturbance, both from iceberg scour^28^ and predation by invertebrates^27^, and where the availability of hard substrate is a major structural factor^29,30^, competitive exclusion, rather than coexistence, would have been the expected outcome for these two coral morphs.

**Fig. 2:**
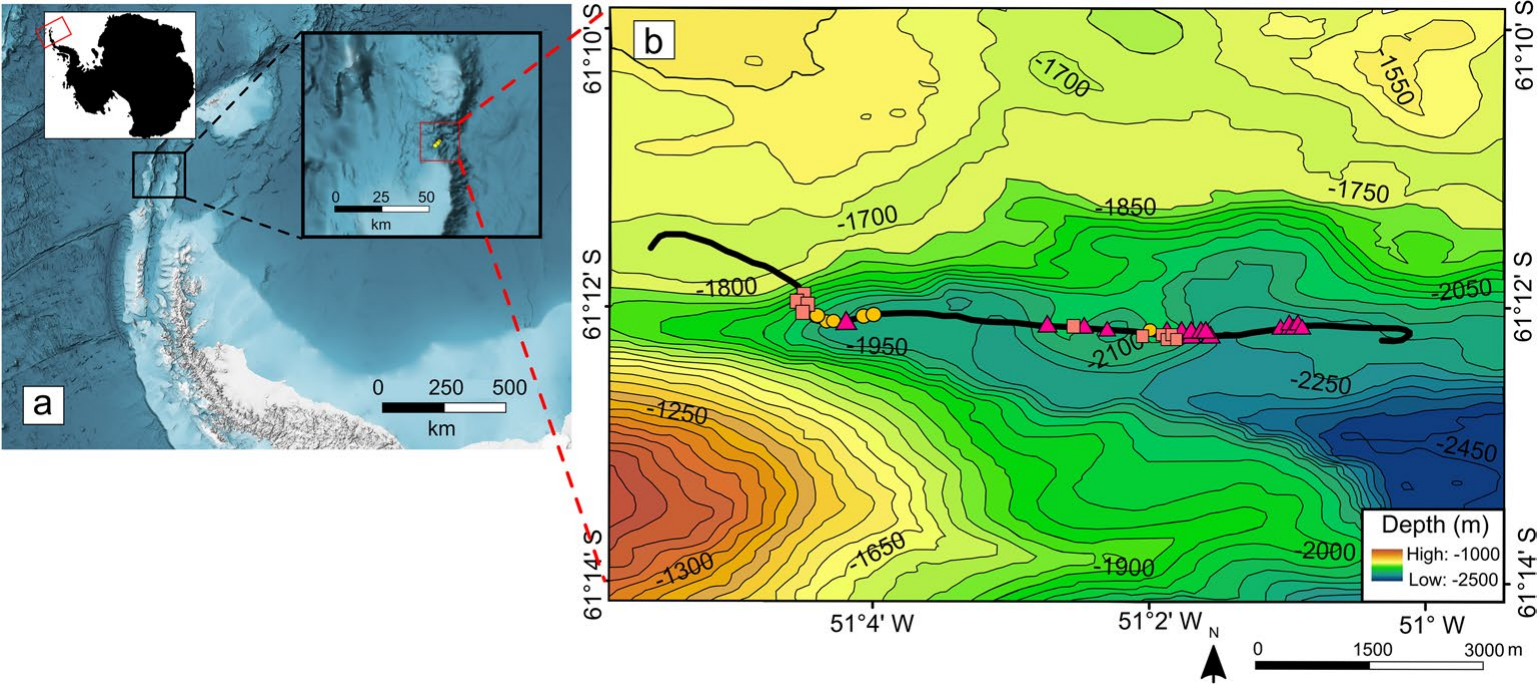
(**a**) Location of the Powell Basin. (**b**) Bathymetry of the Powell Basin (modified from ref^35^). Black line refers to PS118 Profile 69-1 cruise track, with symbols referring to sites analysed in this study.

Here, we study the population ecology of these two coral colour morphs in the Powell Basin (Fig. 2), using Spatial Point Process Analysis (SPPA) to investigate whether there is interaction between the two morphotaxa, or whether coexistence is entirely due to random effects. For sessile organisms, seabed photographs on hard substrates provide a near census record of abundance and capture their positions and spatial distributions. These positions are important because organisms are rarely randomly distributed in space, and spatial statistics can reveal the underlying processes behind the spatial distributions^31,32^. Corals are usually only identifiable by skeletal characteristics, however, from the resolution of seabed photographs (Fig. 1), septa and calices are not well defined. Comparison with published records of scleractinians in Antarctic and sub-Antarctic waters near the Antarctic Peninsula^33,34^ suggest that the Powell Basin cup corals are likely of the genera *Caryophyllia* Lamarck, 1801, or *Flabellum* Lesson, 1831. Rounded calices and conical corallum on some dead specimens suggest that they may be *Caryophyllia,* however, some specimens with triangular columella and flabellate corallum more closely resemble *Flabellum* (Schejter 2024, personal communication). At the resolution of the data available, skeletal morphology of the cup corals cannot be definitively assigned to species or genus level taxonomic identification, and the lack of physical samples prohibits genomic identification. As such, in these photographs, these very similar morphs are distinguishable only by the colour of their tentacles, which can be either orangey-red (the “orange” morph) or a pale pink (the “pink” morph) (Fig. 1). To ensure we are clear on the level of taxonomic certainty we refer to them as “morphs” without assigning a genus name.

We use cup corals as the study organism as they are solitary (enabling accurate population counts), abundant (enabling model fitting), and present in two morphs (enabling direct pairwise comparison). In SPPA, each individual is treated as a point, and a pair correlation function (PCF) describes how the density of the points (here, cup corals) change over the sampled area (here, photographs). Only four processes can influence the spatial positions of sessile individuals: (i) interactions with their environment, such as response to substrate type, slope, or food delivery^31,32^, (ii) reproductive processes, (iii) interactions such as facilitation and competition between and within members of the community, and (iv) mortality processes (such as through predation, parasitism and disease)^31,32^, thus SPPA can be used to link the spatial pattern to a biological process. The power of SPPA in fitting patterns to process has demonstrated significant utility in inferring biological processes even when they cannot be directly observed, e.g., habitat preferences of trees^36,37^, octocorals^38^, and sponges^38^; pathogen and disease-related sponge^38^ and coral^39,40^ mortality; dispersal limitations of seeds^36,37,41^; competition for resources by trees^42^ and by octocorals^38^; facilitation between woody plants and ectomycorrhizal fungi^43,44^, and between octocorals and sponges^38^.

The simplest scenario within SPPA is that the organisms’ PCFs display complete spatial randomness (CSR), which is modelled as a homogenous Poisson model^31^. When CSR best describes an observed PCF, there are no biotic or abiotic processes that significantly affect the population at the studied spatial scales. Non-CSR distributions of the population can show patterns of significant aggregation or significant segregations at different spatial scales, with non-CSR patterns corresponding to different underlying processes. At a particular spatial scale, when the PCF = 1, the taxon displays CSR, when PCF > 1, the individuals within the taxon are significantly aggregated, and when PCF < 1, individuals within the taxon are significantly segregated. The Diggle’s Goodness-of-fit test^45^ assesses how well the PCF fits the simulated model, where *p*_*d*_ = 0 corresponds to a bad fit to CSR, and *p*_*d*_ = 1 corresponds to a perfect fit to CSR.

Patterns of aggregation or segregation may be caused by an environmental preference, such as substrate type (here, elevated boulders or raised seafloor, flat rocks, and debris-filled gulleys), which can be modelled by a Heterogenous Poisson process^32,36^. Spatial distributions may also be affected by reproductive processes, such as where offspring surround their parent^46^, and these are best modelled by a Thomas Cluster process for a single, dispersal limited reproductive event^31,36^. Spatial distributions may also be affected by a combination of process, such as the combination of reproductive processes and substrate preference.

We fit these four different models to our observed spatial patterns in order to identify which underlying biological processes, such as reproduction, niche separation, and competition, are most likely responsible for shaping the distribution of the two cup coral morphs, and the parameters relevant to these processes in our study.

## Results

### Coral abundance and community type

**Fig. 3:**
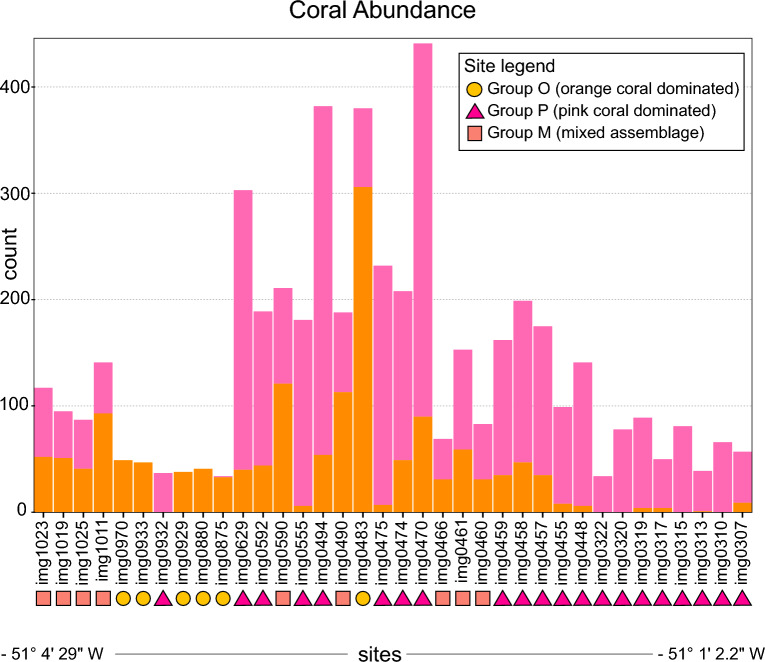
The number of corals in each analysed site. Bars are sorted in order of longitude, but not to scale. Orange bars refer to orange corals, pink bars refer to pink corals. Dots refer to sites in Group O; triangles to sites in Group P; boxes to sites in Group M.

The Powell Basin slope hosts large numbers of solitary cup corals. Within the 36 sites in this study, we identified 3431 pink corals and 1544 orange corals (Fig. 3 and Supplementary Table S1). The minimum number of individual corals (of either colour morph) present at a particular site was 34 (density of 11.1 individuals/m^2^), and the maximum was 441 (density of 144 individuals/m^2^). Based on the proportion of orange corals to pink corals, we split our images into three groups: Group O was dominated by orange corals and consisted of 6 sites, Group P was dominated by pink corals and consisted of 21 sites, and Group M had mixed assemblages with roughly equal proportions of orange and pink corals and consisted of 9 sites (Fig. 3, Supplementary Table S1).

In the orange dominated Group O, five out of six sites had only orange corals. The minimum number of orange corals in a site was 33 (density of 10.8 individuals/m^2^), and the maximum 306 in img0483 (density of 99.9 individuals/m^2^), with a median number of 44 orange corals per site (Fig. 3, Supplementary Table S1). One site (img0483) had 74 pink corals (density of 24.2 individuals/m^2^).

In the pink dominated Group P, thirteen out of twenty one sites had pink corals only. The minimum number of pink corals in a site was 34 (density of 11.1 individuals/m^2^), and the maximum was 351 (density of 114.6 individuals/m^2^), with a median number of 127 pink corals per site (median density of 41.5 individuals/m^2^) (Fig. 3, Supplementary Table S1). Of the eight sites that also had orange corals, the minimum number of orange corals was 35 (density of 11.4 individuals/m^2^), the maximum was 90 (density of 29.4 individuals/m^2^), with a median of 45.5 orange corals in these eight sites (median density of 14.9 individuals/m^2^). The median number of corals per site, regardless of morph type, was 141, with a median density of 46 individuals/m^2^ (Fig. 3, Supplementary Table S1).

In the mixed Group M, all nine sites had approximately equal numbers of orange and pink corals. The minimum number of orange corals was 31 (density of 10.1 individuals/m^2^), the maximum was 121 (density of 39.5 individuals/m^2^), with a median of 52 orange corals per site (median density 17 individuals/m^2^). The minimum number of pink corals was 38 (density of 12.4 individuals/m^2^), the maximum was 94 (density of 30.7 individuals/m^2^), with a median of 52 pink corals per site (median density 17 individuals/m^2^). The median number of corals per site, regardless of morph type, was 117, with a median density of 38.2 individuals/m^2^ (Fig. 3, Supplementary Table S1).

Across the sites, the median density for orange corals was 14.4 individuals per square metre in Group O, 14.9 individuals per square metre in Group P, and 17 individuals per square metre in Group M. Median density for pink corals was 41.5 individuals per square metre in Group P, and 17 individuals per square metre in Group M (Fig. 3, Supplementary Table S1).

### Population ecology

**Fig. 4:**
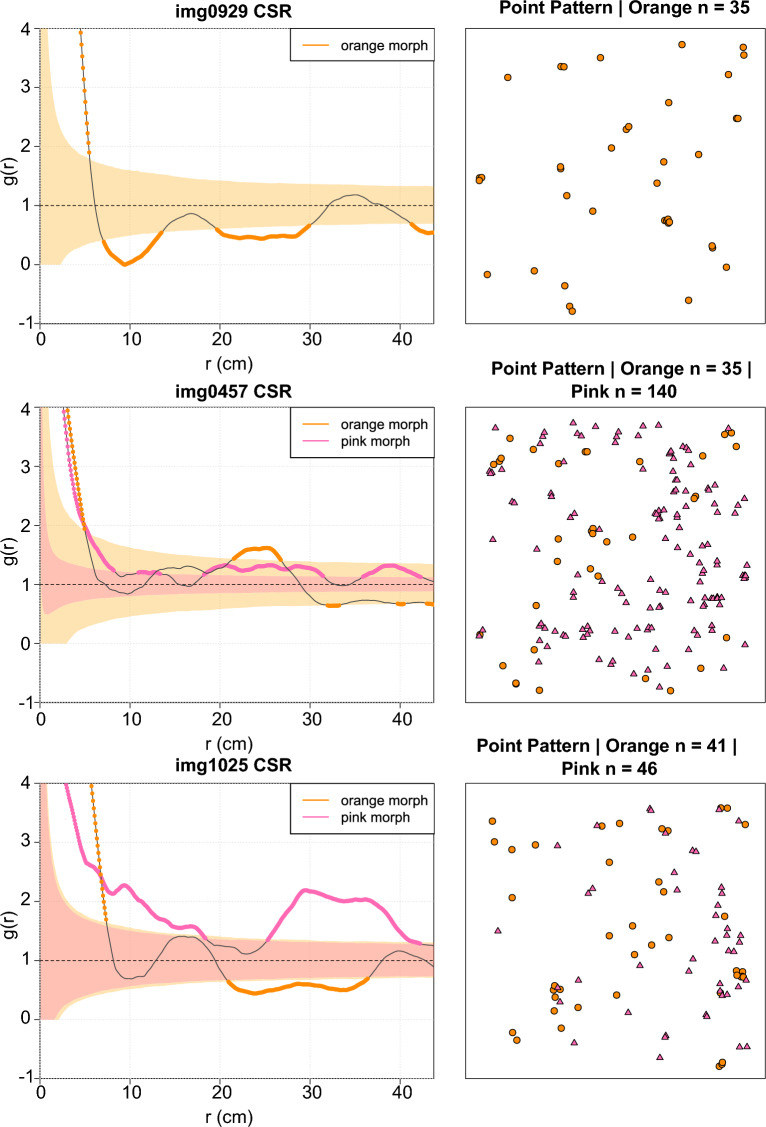
The pair correlation function of three sites (black lines), plotted over a CSR model, with their corresponding point patterns. Orange shaded area refers to simulated CSR for orange corals, and pink shaded area refers to simulated CSR for pink corals. When lines are filled in colour, the pair correlation function is significantly different from CSR.

None of the observed coral patterns displayed complete spatial randomness (*p*_*d*_ value < 0.001, Supplementary Table S1), and instead, were significantly aggregated or segregated at differing spatial scales. For example (Fig. 4), orange corals in site img0929 are significantly aggregated below 7 cm (PCF > 1), and significantly segregated at 7–10 cm, 20–30 cm and at 40–45 cm (PCF < 1), indicated by solid orange lines. In site img0457, orange corals are significantly aggregated below 5 cm and between 22 and 27 cm. The pink corals at this same site are aggregated at almost all spatial scales (pink lines filled in), except at 15–20 cm and 32–35 cm. Contrasting patterns are present between the orange and pink corals in site img1025: the orange corals are significantly aggregated below 10 cm, and segregated at 22–35 cm, whereas the pink corals are significantly aggregated at all spatial scales except 18–25 cm.

**Fig. 5:**
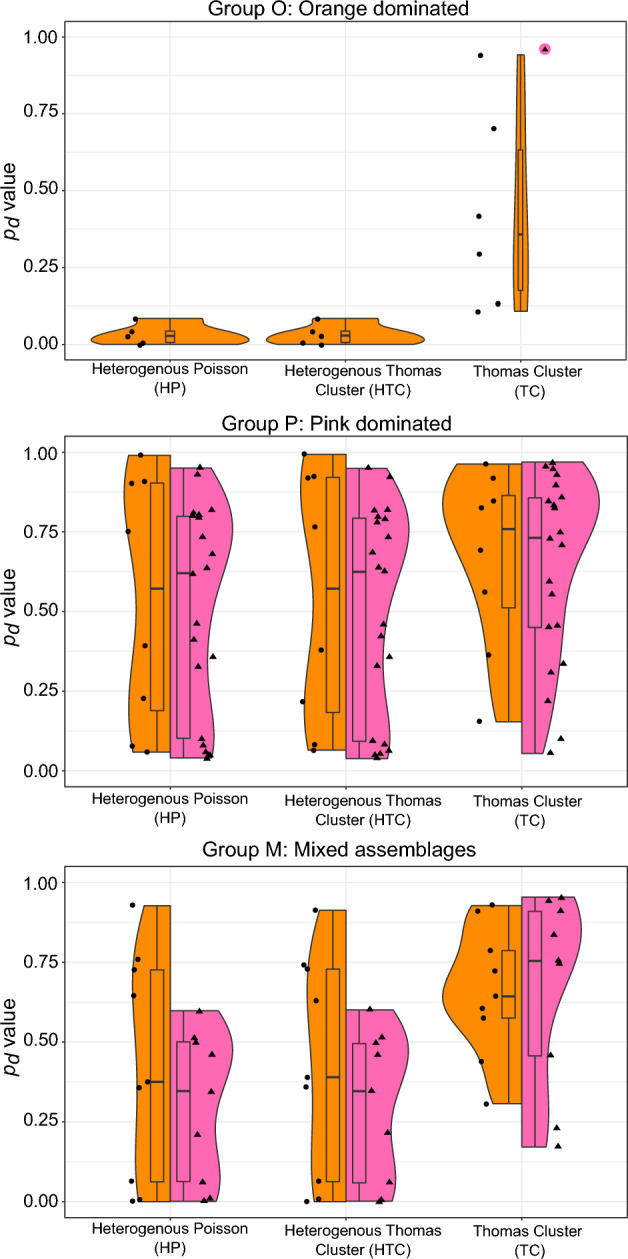
Goodness of Fit values for the 3 non-CSR models in the three groups. Orange violins refer to orange corals, and pink violins refer to pink corals.

Instead of CSR, all populations in this study showed better fits to the three other tested models (Fig. 5, Supplementary Table S1). Orange dominant (Group O) orange corals are best described by a Thomas Cluster process (median *p*_*d*_ value = 0.3576), whereby a parent individual coral is surrounded by offspring. Interactions with substrate (Heterogenous Poisson model), and the mixed effect of dispersal and substrate (Heterogenous Thomas Cluster model), produce lower scores (median *p*_*d*_ value = 0.0277 and median *p*_*d*_ = 0.0287 respectively). When mixed with pink corals (Group M), the PCF is also best described by a Thomas Cluster process (median *p*_*d*_ value = 0.6426, compared to median *p*_*d*_ = 0.3751 for HP and median *p*_*d*_ = 0.3898 for HTC). In pink dominated communities (Group P), orange corals are also best described by TC models, testing dispersal limitations only (median *p*_*d*_ value = 0.7586), compared to HP models (median *p*_*d*_ value = 0.5715) and HTC models (median *p*_*d*_ value = 0.5718) (Fig. 5, Supplementary Table S1).

Pink corals are best described by Thomas Cluster processes both when they are dominant (Group P), and when mixed with orange corals (Group M) (Fig. 5 and Supplementary Table S1). In Group P, the TC fit (median *p*_*d*_ value = 0.7311) provides a better model fit than Heterogenous Poisson (median *p*_*d*_ value = 0.6203) and the Heterogenous Thomas Cluster models (median *p*_*d*_ value = 0.6246). In Group M, the TC model (median *p*_*d*_ value = 0.7539) is a much better fit than HP (median *p*_*d*_ value = 0.3467) and HTC models (median *p*_*d*_ value = 0.3460) (Fig. 5, Supplementary Table S1).

### Reproductive cluster variables

**Fig. 6:**
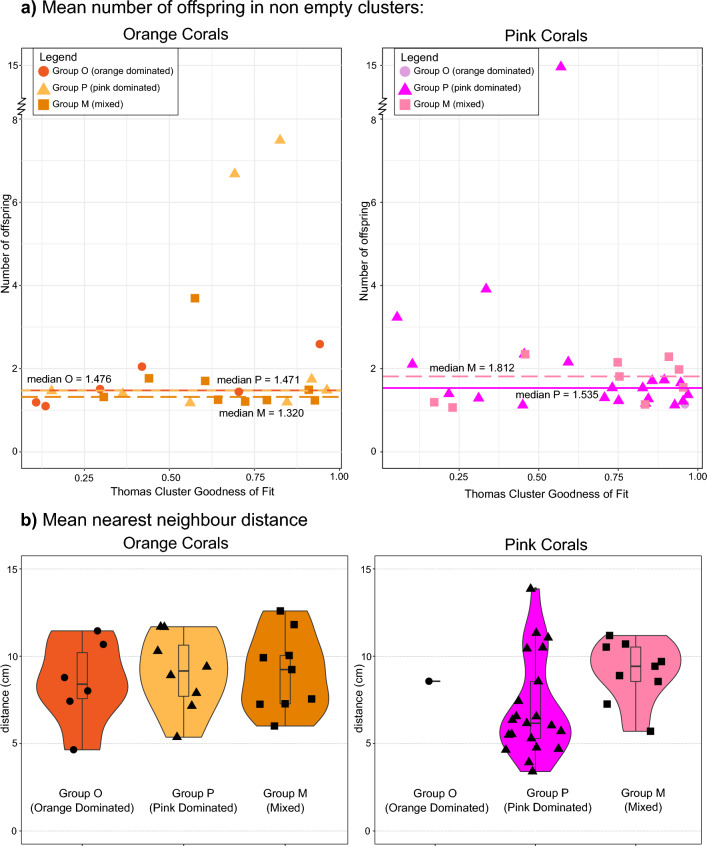
Dispersal parameters across the three group types. (**a**) The mean number of settled offspring per parent coral per site in non-empty clusters. Median values for Group are plotted in dashed lines. (**b**) Mean distances between the corals of the same morph in each site.

As Thomas Cluster models best describe the observed spatial patterns, we investigated reproductive parameters, namely, the mean number of settled offspring per parent coral in a non-empty cluster (Fig. 6a, Supplementary Table S1) in each site, and the probability that an individual coral belongs in a cluster (Supplementary Table S1). Broad patterns show that orange corals do not have any notable changes in their offspring number between Groups O, P, and M (Fig. 6a). They have a median of 1.48 settled offspring per parent coral per site in Group O, 1.48 individuals in Group P, and 1.32 individuals in the mixed Group M. In contrast, pink corals show a notable increase between the number of settled offspring per parent per site in Group P versus in Group M. Pink corals have a median of 1.54 offspring per parent coral per site in Group P, while in the mixed group, they have 1.8 offspring.

In order to assess the extent of clustering within individuals of each population in a site, we computed the distance from each individual coral to its nearest neighbouring individual of the same coral morph, i.e., for each orange coral, we calculated the distance to the nearest orange coral, and for each pink coral, we calculated the distance to the nearest pink coral. For orange corals, the nearest neighbour distances remain consistent between groups. Within each site in Group O, an individual orange coral is, by median, 8.5 cm away from the nearest orange coral (Fig. 6b), in Group P, this median distance is 9.2 cm and in Group M, the nearest orange corals are, by median, 9.2 cm apart.

Pink corals, however, have a significant (Wilcoxon test, *p* = 0.0356) change in their nearest neighbour distances when they are dominant (Group P) versus when they are mixed with orange corals (Group M). The closest neighbouring pink coral from another pink coral in Group P is 6.3 cm away, whereas in Group M, the nearest neighbour distance increases to 9.4 cm, an increase of 49%.

## Discussion

Coexistence of the orange and pink colour morph cup corals on the Powell Basin slope, Weddell Sea, Antarctica, is not due to neutral or random effects, as specific patterns are observed in their spatial distributions. Fewer total corals are present in sites where orange corals are dominant, as well as fewer orange dominant sites (Group O), compared to sites which are dominated by pink corals (median of 44 total corals in the 6 orange dominant Group O sites, versus median of 141 corals in the 21 pink dominant Group P sites). The presence of both dominant (where one coral morph dominates in Groups O and P) and mixed sites (where both morphs are present in equal proportions in Group M) suggest some biological process is responsible for structuring the three community types, such as direct competition, niche segregation or dispersal dynamics. As substrate availability is one of the most important factors that determine the spatial structure of Antarctic benthos at most spatial scales (broad, > 60 km; meso, 10–60 km; small, 2–10 km)^47^, we tested for habitat suitability by approximating substrate type within photographs. At our very fine observed spatial scale (0–50 cm) substrate did not appear to structure these distributions, as neither coral morph showed any significant relationship with the proportion of boulders, debris filled gulleys, or flat bedrock, as demonstrated by poor fits to Heterogenous Poisson (HP) models. In the absence of evidence for habitat affiliations (and consequently, niche segregation), it is reasonable to assume dispersal processes and/or competition structured these populations at centimetre to half-metre scales.

With nearest neighbour distances ranging between 6 and 10 cm, we infer that the cup corals in the Powell Basin produce offspring which crawl away or have lecithotrophic larvae, i.e., their offspring are non-planktotrophic. Dispersal distances on this order may be a beneficial adaptation by brooders to a habitat with low disturbance^48^. While it is impossible to assign a species name to our observed cup corals from photographic samples only, histological analyses on *Flabellum* cup corals from the West Antarctica Peninsula show that they have large oocyte sizes, indicating lecithotrophy, and that planulae lack long cilia, indicating demersal crawling rather than swimming larvae post brooding^49^. Crawl away larvae are also known from other cup corals, e.g., *Balanophyllia elegans*^50^ and lecithotrophy is known from deep sea *Caryophillia* from the NE Atlantic^51^. The average number of successfully settled offspring per parent cup coral in the Powell Basin are between 1.3 and 2 individuals, which provides further evidence for brooded larvae which crawl away and settle near their parents. These numbers are consistent with tank experimental observations on the brooding *Caryophyllia huinayensis* from Chilean waters, which showed that over a period of 3.1 years, from seven reproductive polyps, only 19 larvae successfully settled from a total of 1647 planulae^48^. Brooded larvae also have high energy requirements, so few are produced at a time—histological analyses on *Balanophyllia malouinensis* from Burdwood Bank in the Drake Passage found between 10 and 20 larvae in the mesenteries of females^52^, compared to hundreds to thousands of gametes for broadcast spawning solitary corals^53^.

The consequence of this mode of reproduction is that offspring settle close to a parent that has already found a suitable local habitat that maximises resources^49^. This tendency explains the high density of cup corals within sites (medians ranging from 14 to 41 individuals per square metre), however, on a larger spatial scale, the distribution of cup corals on the Powell Basin slope is patchy, with a likely low overall mean density (Fig. 1).

Of the two coral morphs in the Powell Basin, we infer orange corals to be the stronger competitor because the reproductive behaviour of the orange morph remains consistent across all three group types: the number of settled offspring, as well as dispersal distances, show no significant change when orange corals are dominant in Group O, when they are the minority morph in Group P, and when they are mixed with pink corals in Group M. In contrast, pink corals exhibit a change in reproductive behaviour when they are mixed with orange corals in Group M. Despite the higher energy requirements, pink corals have a significant increase in their dispersal distances, increasing from 6.3 cm when they are dominant (suggesting that this is the optimal distance), to 9.4 cm when they are mixed, an increase of 1.5 times. There is also a notable increase in settled offspring number, where in Group P, pink corals have a median of 1.54 settled offspring per parent coral, while in the mixed group M, they have 1.8 settled offspring. The population size of pink corals also changes when they are dominant (median of 127 individuals per site in Group P) versus when they are sympatric to orange corals (median of 52 individuals per site in Group M) and are almost excluded in orange dominance (with only one site in Group O having pink corals as well), demonstrating a significant reduction in abundance, as a result of interspecific competition. The robustness of the orange corals regardless of group type, their presence across all three groups, and the changing behaviour of the pink corals imply that without this adaptability, pink corals would be the losers in the competition.

Therefore, the coexistence of the two coral morphs on the Powell Basin slope is likely due to the greater adaptability and dispersal capability of the pink coral morph, instead of neutral and random dispersal. Both of these factors may explain the observed distribution of cup corals in this ecosystem: pink corals are twice as abundant as orange corals (3431 pink corals versus 1544 orange corals) and have a much greater geographic spread in the Powell Basin (21 pink dominant sites versus 6 orange dominant sites). As planulation is the only motile phase for sessile taxa, which would allow for spatial distributions to change, increasing offspring number and dispersal distance may be a strategy for these pink corals to disperse out of unfavourable environments^54^. It is possible that stepping-stone style reproductive events over several generations are likely responsible for the wider geographic range observed for the pink corals.

In this study we have inferred that for cup corals in the Powell Basin, competition and dispersal plasticity allows for coexistence. The Powell Basin epibenthos is composed primarily of sessile suspension and filter feeding organisms^27^, against which the cup corals likely also compete, but we found no direct relationship in abundance with these other ecological groups^27^. The dominant taxonomic group, stylasterid corals, inferred to also have brooded, crawling larva^55^ are notable for their ubiquity in this system, suggesting that their larvae are highly successful at settling, and possibly have large numbers of offspring as they are dense and widespread. Other observed sessile taxa, such as actiniarians, octocorals (*Anthomastus* and *Alcyonium)*, and gorgonians (*Echinisis* and *Thouarella)* have patchy, but dense distributions in the Powell Basin (see Fig. 3a from ref^27^). These local patches are likely also caused by dispersal plasticity and stepping stone-like dispersal events^54^. Patchiness driven by dispersal plasticity likely enables taxa with similar ecological functions to coexist, thereby increasing alpha diversity in Antarctic deep sea hard substrate communities.

While we only studied cup corals here, the dispersal processes we resolved may also be responsible for the high alpha diversity and high abundance of the Powell Basin^27^. Our results show that, in contrast with comparable Antarctic shallow water systems, which are heavily structured by physical drivers such as ice scour^56^, biological processes can play an important role in deep water community ecology.

## Methods

### Data collection

Seabed photographs of the Powell Basin were collected in order to survey a rarely sampled region, and were taken by the Ocean Floor Observation and Bathymetry System (OFOBS), a towed camera system mounted on the icebreaker RV *Polarstern*^57^, collected during the PS118 expedition between April–May 2019^35,58^. Still images of 26-megapixel resolution were recorded throughout each deployment of the OFOBS, with photographs taken every 20 s. To localize the collected data, the OFOBS was mounted with a Posidonia transponder for ultra-short baseline triangulation. In optimal circumstances, each photograph had three laser dots placed in an equilateral triangle (50 cm sides) near the centre of the photograph to enable spatial scaling and had both the geographical coordinates and the water depth at which the photograph was taken. Profile 69-1^59^ imaged the western flanks of the Powell Basin, collecting 2723 photographs. Of these photographs, 1073 did not contain GPS metadata due to Posidonia malfunctioning, and a further 496 were fully black (either because they were taken during ascent/descent, or due to the flash not illuminating). Removing these photographs left 1154 photographs for our analyses.

### Photo data annotation

Due to variable bathymetry, local swells and challenging operational conditions, distance from the seafloor to the imaging platform was not consistent, therefore aerial coverage of each photograph differed on the metre scale. In order to maximize photo quality and localize our results, we manually selected optimal photographs which fit several parameters: (i) contained GPS metadata, (ii) were photographed roughly parallel to the seafloor, (iii) were optimally lit, (iv) were taken $\lessapprox$ 4 m above the ground. These parameters help position the photo in space, minimize size distortion of observed features, and enable us to provide as accurate identifications as possible from image data only.

We used Inkscape, Version 1.1.1^60^ to annotate the photographs, where we scaled each photograph using the three laser dots (c.f. ref^27^). We applied a sample box of 1.75 m × 1.75 m to each sampled photograph, providing a coverage area of 3.06 m^2^ per photo. The fifth parameter used for photo selection was that the sampled area contained at least 30 individual solitary cup corals, as this is the minimum number of points required to describe an observed spatial pattern^32^. We identified 47 images with abundant numbers of cup corals that fit selection parameters (i)–(iv), but 11 had to be excluded from analyses as they contained fewer than 30 individuals, i.e., did not fit parameter (v), leaving us with a total of 36 images (from here on referred to as “sites”) where the population ecology of cup corals could be studied. For each individual, annotated as ellipses or circles, we collected the local x and y coordinates, and assigned the “pink” or the “orange” colour-morph ID.

In order to test whether cup corals have substrate preferences, we quantified the substrate within the sample area of each site into three different categories: elevated seafloor or boulders, flat rocky sections, and debris-filled gulleys. In line with the Wentworth scale^61^, clasts that measured at least 25 cm in at least one dimension were classified as a “boulder”; smaller pebbles, whether they were colonized by a coral or not, were classified as debris within our “debris-filled gulleys” category. In Inkscape, we drew filled freehand polygons around each of the observed boulders, flat sections and debris-filled gulleys. We exported a raster image of the polygons from Inkscape, which we then read into Fiji^62^, in order to extract the RGB value of every pixel in the sample area.

Analyses were carried out in R, Version 4.2.2^63^, and the code is available on GitHub (https://github.com/Mingmingkhan/competitive-corals). We used a hierarchical cluster analysis in the vegan package^64^ on the proportion of orange and pink corals present in each site (Bray–Curtis distance measure) to split each site into three groups: orange coral dominated, pink coral dominated, and mixed assemblages. We used the R package spatstat version 3.0-8^65^ for all spatial analyses.

### Model fitting

For each site, the point pattern of both the orange and pink corals were quantified using PCFs. The PCFs were calculated from the spatial data using a grid of 0.1 cm cells, with a smoothing done of 5 cm to appropriately manage noise within the observed data^32^. In order to assess whether the observed data deviated from the null model (here CSR), we ran 9999 Monte Carlo simulations on a homogenous Poisson background^31,45^ with the highest and lowest 5% (here 500) simulations removed (cf. ref^32,65^). We tested fit of the observed PCF to the CSR model using Diggle’s Goodness of Fit test^45^ using the R package selectspm^66^, where *p*_*d*_ = 0 corresponds to a bad fit to CSR, and *p*_*d*_ = 1 corresponds to a perfect fit to CSR.

Within a site, when a particular coral morphotype population had excursions outside the simulation envelopes, i.e., did not exhibit CSR, we tested the further three models: (i) heterogenous Poisson models to test interactions with substrate, the “HP model”, (ii) single Thomas Cluster models to test reproductive processes, the “TC model”, and (iii) a mixed heterogenous Poisson and Thomas Cluster model to test the mixed effects of substrate and reproduction, the “HTC model” (Fig. 3). For each tested model, we ran 9999 Monte Carlo simulations, with the 500th lowest and highest values defining the simulation envelope, and assessed model fit using the Diggle Goodness of Fit^45^ as above. For the Thomas Cluster model, we restrict the goodness of fit range to a 10 cm and a 20 cm radius, instead of fitting over the full 50 cm range, as all PCFs showed significant aggregations at 10 cm, and so we wanted to focus on the aggregated patterns. For each site, we also extracted the mean number of settled offspring in a non-empty cluster, and the probability that an individual coral belongs in a cluster, which are both outputs from the Thomas Cluster model fit^65^.

## Supplementary Information

Supplementary Information.

## Data Availability

Seabed photographs used in this study can be found on PANGAEA: https://doi.pangaea.de/10.1594/PANGAEA.918924. All code and data used in the study can be found on GitHub: https://github.com/Mingmingkhan/competitive-corals/. Summarized results are available in Supplementary Material.

## References

[1] Begon, M., Townsend, C. R. & Harper, J. L. *Ecology: From Individuals to Ecosystems* (Blackwell, 2012).

[2] Volterra, V. Fluctuations in the abundance of a species considered mathematically. *Nature* **118**, 558–560. 10.1038/118558a0 (1926).

[3] Lotka, A. J. The growth of mixed populations: Two species competing for a common food supply. *J. Wash. Acad. Sci.* **22**, 461–469 (1932).

[4] Taniguchi, Y. & Nakano, S. Condition-specific competition: Implications for the altitudinal distribution of stream fishes. *Ecology* **81**, 2027–2039. 10.1890/0012-9658(2000)081[2027:CSCIFT]2.0.CO;2 (2000).

[5] Tansley, A. G. On competition between *Galium Saxatile* L. (*G. Hercynicum* Weig.) and *Galium Sylvestre* Poll. (*G. Asperum* Schreb) on different types of soil. *J. Ecol.* **5**, 173. 10.2307/2255655 (1917).

[6] Connell, J. H. The influence of interspecific competition and other factors on the distribution of the barnacle *Chthamalus Stellatus*. *Ecology* **42**, 710–723. 10.2307/1933500 (1961).

[7] Frinault, B. A. V. et al. Spatial competition in a global disturbance minimum; The seabed under an Antarctic ice shelf. *Sci. Total. Environ.* **903**, 166157. 10.1016/j.scitotenv.2023.166157 (2023). 37572912 10.1016/j.scitotenv.2023.166157

[8] Chesson, P. Mechanisms of maintenance of species diversity. *Annu. Rev. Ecol. Syst.* **31**, 343–366. 10.1146/annurev.ecolsys.31.1.343 (2000).

[9] Knowlton, N. & Jackson, J. B. C. New taxonomy and niche partitioning on coral reefs: Jack of all trades or master of some?. *Trends Ecol. Evol.* **9**, 7–9. 10.1016/0169-5347(94)90224-0 (1994). 21236753 10.1016/0169-5347(94)90224-0

[10] Vermeij, M. J. A., Sandin, S. A. & Samhouri, J. F. Local habitat distribution determines the relative frequency and interbreeding potential for two Caribbean coral morphospecies. *Evol. Ecol.* **21**, 27–47. 10.1007/s10682-006-9122-z (2007).

[11] Bongaerts, P. et al. Sharing the slope: depth partitioning of agariciid corals and associated *Symbiodinium* across shallow and mesophotic habitats (2–60 m) on a Caribbean reef. *BMC Evol. Biol.* **13**, 205. 10.1186/1471-2148-13-205 (2013). 24059868 10.1186/1471-2148-13-205 PMC3849765

[12] Ogden, J. C. & Lobel, P. S. The role of herbivorous fishes and urchins in coral reef communities. *Environ. Biol. Fishes* **3**, 49–63. 10.1007/BF00006308 (1978).

[13] McClanahan, T. R., Kamukuru, A. T., Muthiga, N. A., Yebio, M. G. & Obura, D. Effect of sea urchin reductions on algae, coral, and fish populations. *Conserv. Biol.* **10**, 136–154. 10.1046/j.1523-1739.1996.10010136.x (1996).

[14] McCook, L., Jompa, J. & Diaz-Pulido, G. Competition between corals and algae on coral reefs: A review of evidence and mechanisms. *Coral Reefs* **19**, 400–417. 10.1007/s003380000129 (2001).

[15] Karlson, R. H. & Hurd, L. E. Disturbance, coral reef communities, and changing ecological paradigms. *Coral Reefs* **12**, 117–125. 10.1007/BF00334469 (1993).

[16] Connell, J. H. Disturbance and recovery of coral assemblages. *Coral Reefs* **16**, S101–S113. 10.1007/s003380050246 (1997).

[17] Hughes, T. P. & Connell, J. H. Multiple stressors on coral reefs: A long-term perspective. *Limnol. Oceanogr.* **44**, 932–940. 10.4319/lo.1999.44.3_part_2.0932 (1999).

[18] Bythell, J., Hillis-Starr, Z. & Rogers, C. Local variability but landscape stability in coral reef communites following repeated hurricane impacts. *Mar. Ecol. Prog. Ser.* **204**, 93–100. 10.3354/meps204093 (2000).

[19] Gardner, T. A., Côté, I. M., Gill, J. A., Grant, A. & Watkinson, A. R. Hurricanes and Caribbean coral reefs: Impacts, recovery patterns, and role in long-term decline. *Ecology* **86**, 174–184. 10.1890/04-0141 (2005).

[20] Hubbell, S. P. Neutral theory in community ecology and the hypothesis of functional equivalence. *Funct. Ecol.* **19**, 166–172. 10.1111/j.0269-8463.2005.00965.x (2005).

[21] Alzate, A. & Hagen, O. Dispersal–diversity feedbacks and their consequences for macroecological patterns. *Philos. Trans. R. Soc. B* **379**, 20230131. 10.1098/rstb.2023.0131 (2024). 10.1098/rstb.2023.0131 PMC11495398 38913062

[22] Vellend, M. Conceptual synthesis in community ecology. *Q. Rev. Biol.* **85**, 183–206. 10.1086/652373 (2010). 20565040 10.1086/652373

[23] Alzate, A., Onstein, R. E., Etienne, R. S. & Bonte, D. The role of preadaptation, propagule pressure and competition in the colonization of new habitats. *Oikos* **129**, 820–829. 10.1111/oik.06871 (2020).

[24] Mouquet, N. & Loreau, M. Community patterns in source-sink metacommunities. *Am. Nat.* **162**, 544–557. 10.1086/378857 (2003). 14618534 10.1086/378857

[25] Aronson, R. B. & Blake, D. B. Global climate change and the origin of modern benthic communities in Antarctica. *Am. Zool.* **41**, 27–39. 10.1093/icb/41.1.27 (2001).

[26] Clarke, A., Aronson, R. B., Crame, J. A., Gili, J.-M. & Blake, D. B. Evolution and diversity of the benthic fauna of the Southern Ocean continental shelf. *Antarct. Sci.* **16**, 559–568. 10.1017/S0954102004002329 (2004).

[27] Khan, T. M. et al. Network analyses on photographic surveys reveal that invertebrate predators do not structure epibenthos in the deep (~2000m) rocky Powell Basin, Weddell Sea, Antarctica. *Front. Mar. Sci.*10.3389/fmars.2024.1408828 (2024).

[28] Dowdeswell, J. A. & Bamber, J. L. Keel depths of modern Antarctic icebergs and implications for sea-floor scouring in the geological record. *Mar. Geol.* **243**, 120–131. 10.1016/j.margeo.2007.04.008 (2007).

[29] Gutt, J. Some, “driving forces” structuring communities of the sublittoral Antarctic macrobenthos. *Antarct. Sci.* **12**, 297–313. 10.1017/S0954102000000365 (2000).

[30] Gutt, J. Antarctic macro-zoobenthic communities: A review and an ecological classification. *Antarct. Sci.* **19**, 165–182. 10.1017/S0954102007000247 (2007).

[31] Illian, J., Penttinen, A., Stoyan, H. & Stoyan, D. *Statistical Analysis and Modelling of Spatial Point Patterns* (Wiley, 2008).

[32] Wiegand, T. & Moloney, K. A. *Handbook of Spatial Point-Pattern Analysis in Ecology* (CRC Press, 2013).

[33] Cairns, S. D. Antarctic and subantarctic scleractinia. In *Biology of the Antarctic Seas XI* Vol. 34 (ed. Kornicker, L. S.) 1–74 (American Geophysical Union, 1983).

[34] Schejter, L., Bremec, C. S. & Cairns, S. D. Scleractinian corals recorded in the Argentinean Antarctic expeditions between 2012 and 2014, with comments on *Flabellum (Flabellum) areum* Cairns, 1982. *Polar Res.* **35**, 29762. 10.3402/polar.v35.29762 (2016).

[35] Dorschel, B. *The Expedition PS118 of the Research Vessel POLARSTERN to the Weddell Sea in 2019*. *Berichte zur Polar- und Meeresforschung = Reports on Polar and Marine Research,* 149 pages (2019). 10.2312/BZPM_0735_2019.

[36] Wiegand, T., Gunatilleke, S., Gunatilleke, N. & Okuda, T. Analyzing the spatial structure of a Sri Lankan tree species with multiple scales of clustering. *Ecology* **88**, 3088–3102. 10.1890/06-1350.1 (2007). 18229843 10.1890/06-1350.1

[37] Lin, Y.-C., Chang, L.-W., Yang, K.-C., Wang, H.-H. & Sun, I.-F. Point patterns of tree distribution determined by habitat heterogeneity and dispersal limitation. *Oecologia* **165**, 175–184 (2011). 20640861 10.1007/s00442-010-1718-x

[38] Mitchell, E. G. & Harris, S. Mortality, population and community dynamics of the glass sponge dominated community “The Forest of the Weird” from the Ridge Seamount, Johnston Atoll, Pacific Ocean. *Front. Mar. Sci.* **7**, 565171. 10.3389/fmars.2020.565171 (2020).

[39] Zvuloni, A. et al. Spatio-temporal transmission patterns of black-band disease in a coral community. *PLoS ONE* **4**, e4993. 10.1371/journal.pone.0004993 (2009). 19337384 10.1371/journal.pone.0004993 PMC2660573

[40] Easson, C. G. et al. Exploring individual- to population-level impacts of disease on coral reef sponges: Using spatial analysis to assess the fate, dynamics, and transmission of *Aplysina* red band syndrome (ARBS). *PLoS ONE* **8**, e79976. 10.1371/journal.pone.0079976 (2013). 24244583 10.1371/journal.pone.0079976 PMC3828202

[41] Seidler, T. G. & Plotkin, J. B. Seed dispersal and spatial pattern in tropical trees. *PLoS Biol.* **4**, e344. 10.1371/journal.pbio.0040344 (2006). 17048988 10.1371/journal.pbio.0040344 PMC1609130

[42] Getzin, S. et al. Spatial patterns and competition of tree species in a Douglas-fir chronosequence on Vancouver Island. *Ecography* **29**, 671–682. 10.1111/j.2006.0906-7590.04675.x (2006).

[43] Dickie, I. A., Schnitzer, S. A., Reich, P. B. & Hobbie, S. E. Spatially disjunct effects of co-occurring competition and facilitation. *Ecol. Lett.* **8**, 1191–1200. 10.1111/j.1461-0248.2005.00822.x (2005). 21352443 10.1111/j.1461-0248.2005.00822.x

[44] Liang, Y., Guo, L.-D., Du, X.-J. & Ma, K.-P. Spatial structure and diversity of woody plants and ectomycorrhizal fungus sporocarps in a natural subtropical forest. *Mycorrhiza* **17**, 271–278. 10.1007/s00572-006-0096-z (2007). 17443354 10.1007/s00572-006-0096-z

[45] Diggle, P., Zheng, P. & Durr, P. Nonparametric estimation of spatial segregation in a multivariate point process: Bovine tuberculosis in Cornwall, UK. *J. R. Stat. Soc. C Appl. Stat.* **54**, 645–658. 10.1111/j.1467-9876.2005.05373.x (2005).

[46] Mitchell, E. G., Kenchington, C. G., Liu, A. G., Matthews, J. J. & Butterfield, N. J. Reconstructing the reproductive mode of an Ediacaran macro-organism. *Nature* **524**, 343–346. 10.1038/nature14646 (2015). 26237408 10.1038/nature14646

[47] Gutt, J. et al. Benthic communities and their drivers: A spatial analysis off the Antarctic Peninsula. *Limnol. Oceanogr.* **64**, 2341–2357. 10.1002/lno.11187 (2019).

[48] Heran, T. et al. Life cycle of the cold-water coral *Caryophyllia huinayensis*. *Sci. Rep.* **13**, 2593. 10.1038/s41598-023-29620-x (2023). 36788320 10.1038/s41598-023-29620-x PMC9929098

[49] Waller, R. G., Tyler, P. A. & Smith, C. R. Fecundity and embryo development of three Antarctic deep-water scleractinians: *Flabellum thouarsii*, *F. curvatum* and *F. impensum*. *Deep-Sea Res.* **II**(55), 2527–2534. 10.1016/j.dsr2.2008.07.001 (2008).

[50] Altieri, A. H. Settlement cues in the locally dispersing temperate cup coral *Balanophyllia elegans*. *Biol. Bull.* **204**, 241–245. 10.2307/1543595 (2003). 12807701 10.2307/1543595

[51] Waller, R. G., Tyler, P. A. & Gage, J. D. Sexual reproduction in three hermaphroditic deep-sea *Caryophyllia* species (Anthozoa: Scleractinia) from the NE Atlantic Ocean. *Coral Reefs* **24**, 594–602. 10.1007/s00338-005-0031-3 (2005).

[52] Pendleton, A., Hartill, E. & Waller, R. Notes on reproduction in the deep-sea cup coral *Balanophyllia malouinensis* (Squires 1961) from the Southern Ocean. *Polar Biol.* **44**, 977–986. 10.1007/s00300-021-02854-z (2021).

[53] Waller, R. G. & Tyler, P. A. Reproductive patterns in two deep-water solitary corals from the north-east Atlantic—*Flabellum alabastrum* and *F. angulare* (Cnidaria: Anthozoa: Scleractinia). *J. Mar. Biol. Assoc. UK* **91**, 669–675. 10.1017/S0025315410000822 (2011).

[54] Campana, J. L. M. et al. Dispersal plasticity driven by variation in fitness across species and environmental gradients. *Ecol. Lett.* **25**, 2410–2421. 10.1111/ele.14101 (2022). 36198081 10.1111/ele.14101 PMC9827879

[55] Waller, R. G., Goode, S., Tracey, D., Johnstone, J. & Mercier, A. A review of current knowledge on reproductive and larval processes of deep-sea corals. *Mar. Biol.* **170**, 58. 10.1007/s00227-023-04182-8 (2023).

[56] Zwerschke, N., Morley, S. A., Peck, L. S. & Barnes, D. K. A. Can Antarctica’s shallow zoobenthos ‘bounce back’ from iceberg scouring impacts driven by climate change?. *Glob. Change Biol.* **27**, 3157–3165. 10.1111/gcb.15617 (2021). 10.1111/gcb.15617 33861505

[57] Knust, R. Polar research and supply vessel POLARSTERN operated by the Alfred-Wegener-Institute. *JLSRF* **3**, A119. 10.17815/jlsrf-3-163 (2017).

[58] Purser, A. et al. Seabed video and still images from the northern Weddell Sea and the western flanks of the Powell Basin. *Earth Syst. Sci. Data* **13**, 609–615. 10.5194/essd-13-609-2021 (2021).

[59] Purser, A., Hehemann, L., Dreutter, S., Dorschel, B. & Nordhausen, A. Seabed photographs taken along OFOBS profile PS118_69-1 during RV POLARSTERN cruise PS118. Alfred Wegener Institute, Helmholtz Centre for Polar and Marine Research, Bremerhaven, PANGAEA. (2020). 10.1594/PANGAEA.918924

[60] Inkscape Developers. Inkscape Project (2021).

[61] Wentworth, C. K. A scale of grade and class terms for clastic sediments. *Geol. J.* **30**, 377–392 (1922).

[62] Schindelin, J. et al. Fiji: An open-source platform for biological-image analysis. *Nat. Methods* **9**, 676–682. 10.1038/nmeth.2019 (2012). 22743772 10.1038/nmeth.2019 PMC3855844

[63] R Core Team. *R: A Language and Environment for Statistical Computing* (R Foundation for Statistical Computing, 2022).

[64] Oksanen, J. *et al.* vegan: Community Ecology Package.

[65] Baddeley, A., Rubak, E. & Turner, R. *Spatial Point Patterns: Methodology and Applications with R* (Chapman and Hall/CRC Press, 2015).

[66] de la Cruz, M. selectspm: Select Point Pattern Models Based on Minimum Contrast, AIC and Goodness of Fit (2023).

