# Supplementary material for: Competition drives the dispersal dynamics of two cup coral morphs in populations on the Powell Basin slopes, Weddell Sea, Antarctica
Source: Sci Rep. 2025 May 24;15:18121. doi: 10.1038/s41598-025-02282-7 (PMC12103609; doi:10.1038/s41598-025-02282-7)
Supplement: Supplementary file 1 — Supplementary Information. [file 41598_2025_2282_MOESM1_ESM.pdf]

Competition drives the dispersal dynamics of two cup coral morphs in populations on the Powell Basin slopes, Weddell Sea, Antarctica

Tasnuva Ming Khan 1,2,3*, Huw J. Griffiths 2, Nile P. Stephenson 1,3, Rowan J. Whittle 2, Autun Purser 4, Andrea Manica 1, Emily G. Mitchell 1,3
1 Department of Zoology, University of Cambridge, Downing St, Cambridge CB2 3EJ, United Kingdom
2 British Antarctic Survey, High Cross, Madingley Rd, Cambridge CB3 0ET, United Kingdom
3 University Museum of Zoology, New Museums Site, Downing Street, Cambridge CB2 3EJ, United Kingdom
4 Alfred Wegener Institute, Helmholtz Centre for Marine and Polar Research, Am Handelshafen 12, 23570 Bremerhaven, Germany
*corresponding author: tfmk2@cam.ac.uk

Supplementary Table S1: Number of corals in each image, and the Goodness of Fit values for the four tested models.
CSR – Complete Spatial Randomness; HP – Heterogenous Poisson; TC10 – Thomas Cluster, goodness of fit restricted to 10 cm;
TC 20 – Thomas Cluster, goodness of fit restricted to 20 cm; HTC – Heterogenous Thomas Cluster; # Off-spring – the number of individuals in a non-empty cluster;
P (cluster) – the probability of an individual point belonging in a cluster.

	**Orange Corals**									**Pink Corals**								
			**p_d = Goodness of Fit**					**Thomas Cluster Parameters**				**p_d = Goodness of Fit**					**Thomas Cluster Parameters**	
**Image Name**	**coral count**	**coral density**	**CSR**	**HP**	**TC 10**	**TC 20**	**HTC**	**# Off-spring**	***P* (cluster)**	**coral count**	**coral density**	**CSR**	**HP**	**TC 10**	**TC 20**	**HTC**	**# Off-spring**	***P* (cluster)**
**Group O (dominated by orange corals)**																		
img0483	306	99.9	0.0010		0.1081	0.0916		1.19	0.30	74	24.2	0.0010		0.9596	0.9660		1.14	0.24
img0875	33	10.8	0.0010	0.0066	0.4192	0.4198	0.0069	2.05	0.81	1	0.3							
img0880	41	13.4	0.0010	0.0849	0.1359	0.1359	0.0848	1.10	0.17	0	0							
img0929	38	12.4	0.0010	0.0438	0.2960	0.2963	0.0434	1.51	0.59	0	0							
img0933	47	15.3	0.0010	0.0277	0.9420	0.8917	0.0287	2.59	0.90	0	0							
img0970	49	16	0.0010	0.0001	0.7040	0.7086	0.0001	1.44	0.54	0	0							
**medians**	**44**	**14.35**	**0.001**	**0.0277**	**0.3576**	**0.3581**	**0.0287**	**1.4759**	**0.5661**			**0.001**		**0.9596**	**0.966**		**1.1415**	**0.2373**
**Group P (dominated by pink corals)**																		
img0457	35	11.4	0.0010	0.9898	0.5609	0.5650	0.9932	1.18	0.28	140	45.7	0.0010	0.8169	0.9467	0.9423	0.8175	1.66	0.67
img0458	47	15.3	0.0010	0.0588	0.3629	0.3622	0.0649	1.39	0.51	152	49.6	0.0010	0.0512	0.0548	0.0532	0.0520	3.23	0.95
img0459	35	11.4	0.0010	0.2261	0.9630	0.9092	0.2170	1.48	0.57	127	41.5	0.0010	0.6203	0.8567	0.7482	0.6246	1.71	0.70
img0470	90	29.4	0.0010	0.7509	0.6919	0.7013	0.7658	6.68	1.00	351	115	0.0010	0.3291	0.4545	0.4645	0.3278	2.35	0.87
img0474	49	16	0.0010	0.3921	0.9182	0.9028	0.3777	1.74	0.71	159	51.9	0.0010	0.8081	0.8359	0.8277	0.8195	1.16	0.27
img0494	54	17.6	0.0010	0.0767	0.1537	0.1540	0.0819	1.46	0.56	328	107	0.0010	0.0475	0.2175	0.1357	0.0479	1.39	0.51
img0592	44	14.4	0.0020	0.9083	0.8457	0.8567	0.9232	1.19	0.31	145	47.3	0.0010	0.1022	0.3346	0.3345	0.0925	3.91	0.98
img0629	40	13.1	0.0010	0.9017	0.8253	0.8306	0.9199	7.49	1.00	263	85.9	0.0010	0.4605	0.5928	0.4858	0.4580	2.16	0.83
img0307	9	2.9								48	15.7	0.0010	0.7972	0.4498	0.4496	0.7956	1.12	0.21
img0310	0	0								66	21.6	0.0010	0.7988	0.9690	0.9516	0.7783	1.37	0.49
img0313	1	0.3								38	12.4	0.0020	0.6789	0.7511	0.7408	0.6838	1.23	0.35
img0315	0	0								81	26.4	0.0010	0.7327	0.8947	0.8437	0.7320	1.72	0.70
img0317	4	1.3								46	15	0.0010	0.9502	0.9536	0.9588	0.9492	1.21	0.33
img0319	4	1.3								85	27.8	0.0010	0.4141	0.7311	0.6551	0.4211	1.54	0.61
img0320	0	0								78	25.5	0.0010	0.9287	0.8445	0.8133	0.9248	1.27	0.40
img0322	0	0								34	11.1	0.0760	0.3558	0.9275	0.9562	0.3558	1.12	0.21
img0448	6	2								135	44.1	0.0010	0.0574	0.1027	0.0897	0.0619	2.10	0.82
img0455	7	2.3								91	29.7	0.0010	0.8016	0.8270	0.7870	0.7930	1.53	0.60
img0475	7	2.3								225	73.5	0.0010	0.0405	0.7069	0.6522	0.0386	1.30	0.42
img0555	6	2								175	57.1	0.0010	0.6349	0.5660	0.0021	0.6370	14.95	1.00
img0932	0	0								37	12.1	0.0010	0.0782	0.3111	0.3116	0.0814	1.29	0.41
**medians**	**45.5**	**14.85**	**0.0010**	**0.5715**	**0.7586**	**0.7660**	**0.5718**	**1.4714**	**0.5637**	**127**	**41.5**	**0.001**	**0.6203**	**0.7311**	**0.6551**	**0.6246**	**1.53452**	**0.6045**
**Group M (mixed group)**																		
img0460	31	10.1	0.0010	0.0622	0.3070	0.3064	0.0634	1.32	0.44	52	17	0.0010	0.3467	0.1714	0.1685	0.3460	1.19	0.31
img0461	59	19.3	0.0010	0.9270	0.4398	0.4423	0.9128	1.77	0.72	94	30.7	0.0010	0.4594	0.4569	0.4571	0.4586	2.35	0.87
img0466	31	10.1	0.0010	0.3751	0.5750	0.5790	0.3898	3.69	0.97	38	12.4	0.0010	0.0636	0.2291	0.2287	0.0593	1.07	0.12
img0490	113	36.9	0.0010	0.7259	0.7866	0.7112	0.7285	1.25	0.37	75	24.5	0.0010	0.0012	0.8343	0.8439	0.0020	1.14	0.23
img0590	121	39.5	0.0010	0.6462	0.9098	0.9205	0.6296	1.49	0.58	90	29.4	0.0010	0.5981	0.7539	0.7276	0.6008	1.81	0.74
img1011	93	30.4	0.0010	0.3580	0.9275	0.9553	0.3588	1.24	0.36	48	15.7	0.0170	0.5005	0.7481	0.7746	0.4955	2.15	0.83
img1019	51	16.7	0.0010	0.0004	0.6426	0.6314	0.0003	1.25	0.37	44	14.4	0.0010	0.0089	0.9541	0.9493	0.0082	1.55	0.61
img1023	52	17	0.0010	0.7584	0.7227	0.7317	0.7397	1.21	0.33	65	21.2	0.0080	0.5152	0.9091	0.9467	0.5132	2.29	0.86
img1025	41	13.4	0.0010	0.0069	0.6051	0.6080	0.0069	1.70	0.69	46	15	0.0010	0.2121	0.9409	0.9490	0.2147	1.98	0.79
**medians**	**52**	**17**	**0.001**	**0.3751**	**0.6426**	**0.6314**	**0.3898**	**1.32**	**0.4419**	**52**	**17**	**0.001**	**0.3467**	**0.7539**	**0.7746**	**0.346**	**1.8122**	**0.737**
